# Current News

**Published:** 1895-06

**Authors:** 


### Current News.

### CHICAGO DENTAL SOCIETY.

The following are the officers of the Chicago Dental Society for 1895:

President, W. V. B. Ames; First Vice-President, C. E. Bentley; Second Vice-President, J. A. Dunn; Secretary, A. H. Peck, 65 Randolph Street; Corresponding Secretary, H. A. Costner; Treasurer, E. D. Swain; Librarian, H. A. Gunther.

*Board of Directors.*—G. H. Cushing, *chairman*, J. N. Crouse, J. G. Reid.

*Board of Censors.*—D. C. Bacon, *chairman*, H. W. Sale, E. R. Carpenter.

*Committee on Exhibits.*—G. B. Perry, *chairman.*

H. A. Costner,
*Corresponding Secretary.*

### MISSOURI STATE DENTAL ASSOCIATION.

The Thirty-first Annual Meeting of the Missouri State Dental Association will be held at Pertle Springs, July 9 to 12, 1895, inclusive. All dentists in Missouri are especially invited to attend, and a cordial invitation is extended to those of other States. It is expected that this will be one of the most interesting meetings in the history of the Association.

W. M. Carter,
*Corresponding Secretary.*

Sedalia, Mo.

### NATIONAL ASSOCIATION OF DENTAL EXAMINERS.

The next meeting will be held in the parlors of the Hotel Columbia, Asbury Park, N. J., on Monday, August 5, at ten A.M., and at other times during the week as becomes necessary between the sessions of the American Dental Association. It is important that every State Board be represented. Applications from Boards not in membership will receive immediate attention.

CHARLES A. MEEKER, D.D.S.,
*Secretary.*

29 FULTON STREET, NEWARK, N. J.

---

### PENNSYLVANIA STATE DENTAL EXAMINING BOARD.

The Pennsylvania State Dental Examining Board will hold its next annual meeting at Eagle's Mere, Pa, July 9, 1895.

W. E. MAGILL, Erie, Pa.,
*President.*
J. C. GREEN, West Chester, Pa.,
*Secretary.*

---

### NEW JERSEY STATE DENTAL SOCIETY.

The Twenty-fifth Annual Meeting (the silver anniversary) of the New Jersey State Dental Society will be held in the "Auditorium," Asbury Park, commencing Thursday, August 1, at ten A.M., and continuing through Friday, Saturday, and Monday, closing in time for the meeting of the American Dental Association, commencing Tuesday, August 6, at ten A.M.

The "Auditorium" is the ideal place for holding a summer dental meeting, being situated in the middle of an entire block fronting the surf, with large windows opening from every side in one continuous row, thirty large windows with north light for clinics, and 390 x 25 feet for exhibits.

A branch of the Asbury Park Post-Office will be established in the "Auditorium," and a bureau for general information, with attendants constantly on hand.

The New Jersey head-quarters will be in the Hotel Columbia, with rates from $2.50 to $3.00 per day; several large hotels have made contracts from $2.50 to $4.00 per day, and smaller hotels from $8.00 to $12.00 per week board.

Full particulars and rates, with map of Asbury Park and a plan of the "Auditorium," will appear in the programme.

CHARLES A. MEEKER, D.D.S.,
*Secretary.*

29 FULTON STREET, NEWARK, N. J.

---

### AMERICAN DENTAL ASSOCIATION.

THE American Dental Association holds its next meeting at Asbury Park, N. J., Tuesday, August 6, 1895. I am requested by the President, Dr. J. Y. Crawford, to give notice that it is the privilege and duty of each State and Local Society to appoint and send delegates to the American Dental Association.

Each State and Local Society which has adopted substantially the same code of ethics as that governing the conduct of members of the American Dental Association is entitled to one representative for every five members and fractional part thereof.

Blank certificates for delegates may be had on application to the Corresponding Secretary.

Many questions of interest will come up for discussion at this meeting, and every Dental Society in the United States should be fully represented in this, our National Convention.

EMMA EAMES CHASE,
*Corresponding Secretary.*

APRIL 27, 1895.

---

### MASSACHUSETTS DENTAL SOCIETY.

THE Thirtieth Annual Meeting of the Massachusetts Dental Society will be held in Boston, at the rooms of the Harvard Dental School, beginning on Wednesday, June 5, at two o'clock, P.M., and continuing through Thursday, June 6. *Mark these dates off your appointment book now,* and plan to be with us. The annual dinner will be at Young's Hotel at six o'clock on Wednesday; tickets $1.50

a plate; ladies invited. There will be a number of interesting clinics and exhibits, which will well repay your attention. The councillors will meet promptly at the same place at 9.30 A.M., Wednesday, to transact the business of the Society, so that the entire time of the Society may be given to papers, discussions, etc.

JOS. KING KNIGHT,
*President.*
EDGAR O. KINSMAN,
*Secretary.*

---

### WOMEN'S DENTAL ASSOCIATION.

THE Third Annual Meeting of the Women's Dental Association was held at the office of Dr. Mary H. Stilwell, 1300 Arch Street, Philadelphia, Saturday evening, March 2, 1895.

The following officers were elected for the ensuing year: Dr. Anna T. Focht, President; Dr. Elizabeth A. Davis, Vice-President; Dr. Emily W. Wyeth, Recording Secretary; Dr. Mary H. Stilwell, Corresponding Secretary; Dr. Matilda Groth, Treasurer.

*Executive Committee.*—Drs. Eliza Yerkes, Bertha Jarret, Maria Lasser, Hannah M. Miller, and Martha C. Corkhill.

Vice-presidents from representative States were re-elected. Total membership, forty-two.

EMILY W. WYETH,
*Recording Secretary.*

